# SARS-CoV-2 Infection Increases the Risk of Muscle Injury in Professional Male Soccer Players—A Retrospective Analysis of the Italian and Spanish Major Leagues

**DOI:** 10.3390/idr15040043

**Published:** 2023-07-26

**Authors:** Sandra Miccinilli, Marco Bravi, Giorgio Conti, Federica Bressi, Silvia Sterzi, Fabio Santacaterina, Massimo Ciccozzi

**Affiliations:** 1 Department of Physical and Rehabilitation Medicine, Fondazione Policlinico Universitario Campus Bio-Medico di Roma, Via Alvaro del Portillo, 200, 00128 Rome, Italy; s.miccinilli@policlinicocampus.it (S.M.); m.bravi@policlinicocampus.it (M.B.); g.conti@policlinicocampus.it (G.C.); f.bressi@policlinicocampus.it (F.B.); s.sterzi@policlinicocampus.it (S.S.); 2 Research Unit of Physical and Rehabilitation Medicine, Università Campus Bio-Medico di Roma, Via Alvaro del Portillo, 200, 00128 Rome, Italy; 3 Department of Movement, Human and Health Sciences, University of Rome “Foro Italico”, 00135 Rome, Italy; 4 Unit of Medical Statistics and Molecular Epidemiology, Università Campus Bio-Medico di Roma, Via Alvaro del Portillo, 200, 00128 Rome, Italy; m.ciccozzi@unicampus.it

**Keywords:** SARS-CoV-2, muscle injury, soccer, football, return to sport

## Abstract

A retrospective cohort study on professional soccer players from the Serie A and LaLiga was conducted to investigate the correlation between SARS-CoV-2 infection and muscle injuries. Players were divided into two groups based on whether they contracted the SARS-CoV-2 infection (C+) or not (C−) during the 2020/2021 season. In the 2019–2020 season, both championships showed a similar number of muscular injuries (MI) between C+ and C− (Serie A: *p* = 0.194; 95% CI: −0.044 to 0.215, LaLiga *p* = 0.915; 95% CI: −0.123 to 0.137). In the 2020–2021 season, C+ had a significantly higher number of MI compared to C− in both championships (Serie A: *p* < 0.05; 95% CI 0.731 to 1.038; LaLiga: *p* < 0.05; 95% CI: 0.773 to 1.054). Multiple linear regression analysis confirmed that belonging to C+ in the season 2020/2021 was the variable that most strongly influenced the probability of having a muscle injury. Survival analysis revealed a hazard ratio of 3.73 (95% CI 3.018 to 4.628) and of 5.14 (95% CI 3.200 to 8.254) for Serie A and LaLiga respectively. We found an association between SARS-CoV-2 infection and increased risk of muscle injury, emphasizing the importance of carefully considering the infection in the decision-making process for returning to sport. Therefore, SARS-CoV-2 infection should be judged as a real injury requiring specific assessment and training programs.

## 1. Introduction

In late December 2019, The World Health Organization (WHO) office in China was informed about novel cases of pneumonia of unknown aetiology detected in the city of Wuhan, Hubei province [1]. Afterwards, a new type of coronavirus, named SARS-CoV-2, was isolated and identified by the Chinese authorities. The coronavirus disease 2019 (COVID-19) caused by SARS-CoV-2 was classified as a pandemic on 11 March 2020 [2] by the World Health Organization (WHO). All countries were involved in surveillance of the disease and all sports that included close contact were temporarily suspended. 

Football (soccer) is a physically demanding sport that requires a high level of neuromuscular readiness, including strength, reactivity, and muscular power [3,4]. The characteristics of this sport could therefore justify the increases in muscle injury rates associated with the increase in the number of official seasonal competitions and weekly training sessions [5,6]. However, the outbreak of the SARS-CoV-2 virus has had a significant impact on professional athletes, even in mild cases, leading to muscle weakness, poor tolerance to physical exercise, periods of inactivity and absence from sporting practice, with a considerable impact also on bio-psychosocial context [7,8,9]. COVID-19 is known to cause severe inflammatory responses, respiratory failure, acute respiratory distress syndrome (ARDS), and bilateral pneumonia [1]; in some cases, these symptoms can continue causing what is called long COVID. It can also affect the musculoskeletal system, causing myalgia and sarcopenia in COVID-19 positive patients [10]. Therefore, the question arises whether COVID-19 may have influenced the number of muscle injuries in elite athletes. Previous studies in the literature have observed that the approximately three months of sports activity suspension due to the lockdown caused an increase in the incidence of injuries in the main European football leagues, including the Bundesliga (Germany) [11], LaLiga (Spain) [12] and Premier League (England) [13]. The only exception is represented by the Italian first division (Serie A) [14], where a non-statistically significant difference was observed between pre- and post-lockdown injuries. However, these studies did not show whether contracting SARS-CoV-2 infection can be considered an additional risk factor for injury occurrence. Only the recent prospective study by Wezenbeek et al. [15] involving three Belgian professional male football teams during the first half of the 2020–2021 season, reported a five-fold higher risk of developing a muscle strain after a SARS-CoV-2 infection. The goal of this study is to further investigate the findings reported by Wezenbeek et al. [15] and to retrospectively examine on a larger sample size, the correlation between SARS-CoV-2 infection and muscle injuries among professional football players from two different championships, Serie A and LaLiga. These two championships were chosen because Serie A was an exception in terms of post-lockdown injury increase, while LaLiga had the most active collaboration in identifying and communicating positive cases and its results were more discordant between pre- and post-lockdown.

However, these studies did not show whether having contracted SARS-CoV-2 infection can be considered an additional risk factor for the occurrence of injuries. Recently only the prospective study by Wezenbeek et al., involving three Belgian professional male football teams, during the first half of the 2020–2021 season reported a five times higher risk of developing a muscle strain after a SARS-CoV-2 infection.

The goal of this study is to deepen the results reported by Wezenbeek et al. and to retrospectively verify, on a larger sample, the correlation between SARS-CoV-2 infection and muscle injuries among professional footballers of two different championship Serie A and LaLiga. The choice of these two different championships was because Serie A represented the exception of post-lockdown injuries increase and both Serie A and LaLiga had the most active collaboration in identifying and communicating positive cases [12]. Our hypothesis is that there is a relationship between muscle injury and previous SARS-CoV-2 infection.

## 2. Materials and Methods

### 2.1. Study Design

A retrospective cohort study was conducted according to the STROBE guidelines [16]. The aim was to identify a potential correlation between SARS-CoV-2 infection and muscle injuries in professional soccer players from the Serie A and LaLiga leagues during the 2019–2020 and 2020–2021 football seasons. The study included all players from Serie A and LaLiga during the two seasons, as well as those who changed league levels during this time period (e.g., from a minor league such as Serie B in Italy to the top national league, Serie A) and those who moved to a foreign team. Athletes were divided into two groups: COVID-positive (C+) and COVID-negative (C−).

The grouping was performed as follows: (1) all football players infected during the 2020–2021 season were classified as “C+”, while all players who did not contract the infection during this time period were classified as “C−”; (2) players who contracted the infection during the 2019–2020 season or during off-season periods (e.g., season summer suspension) were excluded from the study (Serie A n = 29; LaLiga n = 38) and their data were removed from both seasons. This created a database with pre-COVID (2019–2020 season) and post-COVID (2020–2021 season) data.

### 2.2. Data Collection

Data collection was conducted using the Transfermarkt24 site (URL: https://www.transfermarkt.co.uk/, accessed on 12 September 2022) a website founded in Germany in May 2000 which contains a wealth of football information from leagues around the world, including data on the type and severity of injuries. This methodology is consistent with previous studies [17,18,19,20]. The data collected are specified in Table 1.

### 2.3. Statistical Analysis

Statistical analysis was performed according to the CHAMP statement [21] using MedCalc software (Version 20, MedCalc Software Ltd., Ostend, Belgium). The normal distribution of the data was verified using the Kolmogorov–Smirnov test. The comparison of mean injuries between the two groups in the 2019–2020 and 2020–2021 seasons (i.e., change between seasons) was performed using a parametric t-Student test. A multiple linear regression analysis was carried out in both seasons to analyse the relationship between the dependent variable, muscle injuries, and the independent variables of age, SARS-CoV-2 infection, minutes played, and matches played. Additionally, a Kaplan–Meier survival analysis was performed, using the elapsed time (in days) from the start of each championship to the day on which the injury occurred during the in-season period as the “survival time”. If no injuries occurred, the total duration of the season in days was used as the survival time. The Kaplan–Meier curves were analysed using the Logrank Test in both seasons.

## 3. Results

**Figure 1:**
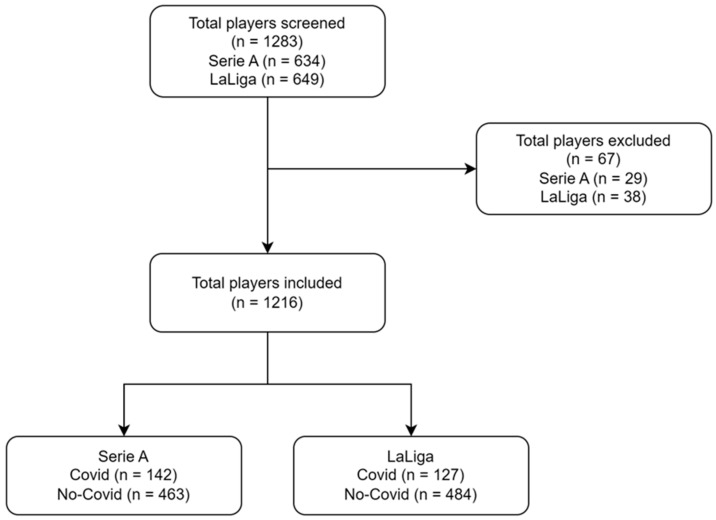
Participants enrolment flowchart.

The study included 634 players from Serie A (mean age = 27.9 ± 4.7 years) and 649 players from LaLiga (mean age = 28.2 ± 4.2 years), for a total of 1283 elite football players. Of the Italian championship players, 171 (27.3%) were infected with SARS-CoV-2, with 29 excluded due to being infected during the 2019-2020 season. In LaLiga, a total of 165 (25.4%) players were infected with SARS-CoV-2, with 38 excluded as they were infected during the 2019–2020 season (Figure 1). 

In the 2019–2020 Serie A championship, on average each player participated in 21 soccer matches with an average of 1502 ± 898 min played. On average group C+ played for 1719 ± 928 min in 23.1 ± 9.7 matches, while group C− played for 1440 ± 879 min in 20.8 ± 9.9 matches, showing a significant difference between the two groups both for minutes played (*p* = 0.001 95% CI: 117.22 to 451.170) and for matches played (*p* = 0.015 95% CI: 0.44 to 4.15). 

In the 2020/2021 season, on average each player participated in 22 soccer matches with an average of 1453 ± 927 min played, group C+ played on average for 1454 ± 912 min in 21.5 ± 10.1 matches, while group C− played for 1456 ± 934 min in 22.12 ± 10.6 matches, showing a non-significant difference between the two groups both for minutes played (*p* = 0.997 95% CI: −173.67 to 174.38) and for matches played (*p* = 0.554 95% CI: −2.57 to 1.38). 

The C+ group reported a mean change between seasons of 1.6 ± 9.8 higher number matches played in the 2019/2020 season (*p* = 0.05; 95% CI: 0.003 to 3.225) and an average of 265 ± 893 more minutes played in the 2019/2020 season (*p* < 0.05; 95% CI: 118.54 to 412.03; the C− group reported a mean change between seasons of 1.2 ± 10.3 higher number matches played in the 2020/2021 season (*p* = 0.008; 95% CI: −2.235 to −0.336) and an average of 16 ± 883 more minutes played in the 2020/2021 season (*p* < 0.05; 95% CI: −98.1 to 64.5).

**Figure 2:**
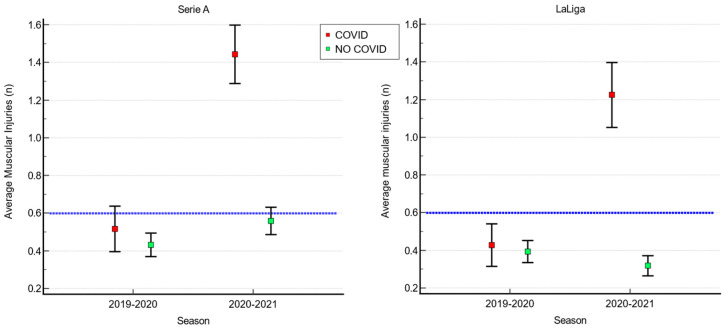
Average number of muscle injuries in the 2019/2020 and 2020/2021 seasons. The dashed blue line represents the average number of injuries per season identified by Bengtsson et al. [22].

In the 2019–2020 Serie A championship, non-significant differences (*p* = 0.194; 95% CI: −0.044 to 0.215) were found in the average number of muscular injuries between the C+ group (0.52 ± 0.75) and the C− group (0.43 ± 0.68). In the 2020–2021 season, the C+ group had a significantly higher number of muscular injuries (1.44 ± 0.96) compared to the C− group (0.56 ± 0.78) (*p* < 0.05; 95% CI 0.731 to 1.038) (Figure 2). The C+ group reported a mean change between seasons of 0.917 ± 1.096 higher number of muscular injuries in the 2020–2021 season (*p* < 0.05; 95% CI: 0.738 to 1.096); the C− group reported a mean change between seasons of 0.131 ± 0.843 higher number of muscular injuries in the 2020–2021 season (*p* < 0.05; 95% CI: 0.05 to 0.209).

The multiple linear regression analysis revealed that, in the 2019–2020 season, muscular injuries were mainly related to age, minutes played, and matches played by the athletes (Table 2). In the 2020–2021 season, in addition to age, belonging to the C+ group was found to be the variable that most strongly influenced the probability of having a muscle injury, unlike the previous season (Table 2).

**Figure 3:**
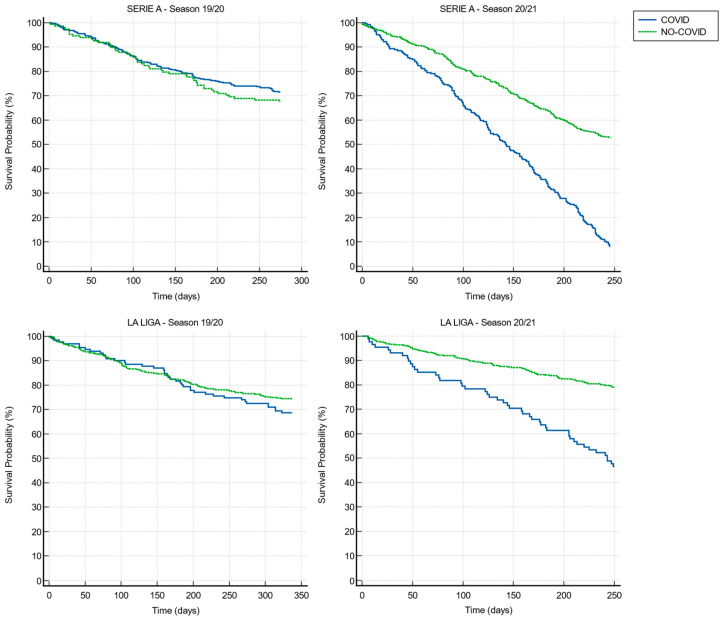
Kaplan–Meier curves relating to muscular injury events of Serie A and LaLiga in the 2019–2020 and 2020–2021 seasons.

The survival analysis based on the Kaplan–Meier curves (Figure 3) revealed that in both groups, the injury rates during the 2019–2020 season did not differ significantly (*p* = 0.401). However, during the 2020–2021 season, the difference in injury rates between the two groups became statistically significant (*p* < 0.05), with a hazard ratio of 3.73 (95% CI 3.018 to 4.628).

In the 2019–2020 LaLiga championship, on average each player participated in 22 soccer matches with an average of 1559 ± 964 min played. On average C+ group played for 1592 ± 929 min in 22.6 ± 11 matches, while group C− played for 1550 ± 974 min in 22.2 ± 10.7 matches, showing a non-significant difference between the two groups both for minutes played (*p* = 0.673 95% CI: −149.36 to 231.33) and for matches played (*p* = 0.688 95% CI: −1.69 to 2.57). 

In the 2020/2021 season, on average each player participated in 23 soccer matches with an average of 1500 ± 921 min played. Group C+ played on average for 1406 ± 873 min in 21.6 ± 9.9 matches, while group C− played for 1525 ± 933 min in 23.1 ± 10.2 matches, showing a non-significant difference between the two groups both for minutes played (*p* = 0.202 95% CI: −299.96 to 63.69) and for matches played (*p* = 0.129 95% CI: −3.57 to 0.45). 

The C+ group reported a mean change between seasons of 1.1 ± 11.1 higher number of matches played in the 2019/2020 season (*p* = 0.273 95% CI: −0.874 to 3.066) and on average 184 ± 969 more minutes played in the 2019/2020 season (*p* < 0.05; 95% CI: 13.139 to 356.31; the C− group reported a mean change between seasons of 0.7 ± 1153 higher number of matches played in the 2020/2021 season (*p* = 0.162; 95% CI: −1.781 to 0.298) and on average 39 ± 1017 more minutes played in the 2019/2020 season (*p* = 0.397; 95% CI: −52.1.1 to 131.2).

In LaLiga, the results indicated that during the 2019-2020 season, there was no significant difference in the average number of muscular injuries between the C+ and C− groups, with 0.40 ± 0.67 and 0.39 ± 0.65 injuries, respectively (*p* = 0.915; 95% CI: −0.123 to 0.137). However, during the 2020-2021 season, the average number of injuries was significantly higher (*p* < 0.05; 95% CI: 0.773 to 1.054) in the C+ group (1.23 ± 1.04) compared to the C− group (0.32 ± 0.59) (Figure 2). The C+ group reported a mean change between seasons of 0.832 ± 1.029 higher number of muscular injuries in the 2020–2021 season (*p* < 0.05; 95% CI: 0.649 to 1.014); the C− group reported a mean change between seasons of 0.131 ± 0.843 lower number of muscular injuries in the 2020–2021 season (*p* < 0.05; 95% CI: −0.144 to −0.005).

The multiple linear regression analysis indicated that in the 2019–2020 season, age was the main factor related to muscle injuries (*p* < 0.05), while games played and minutes played by athletes did not significantly influence the occurrence of injuries. However, in the 2020–2021 season, the group variable had a significant influence on the muscle injury rate (Table 3).

Moreover, in the Spanish championship, the survival analysis (Figure 3) showed how the injuries rates in the 2019–2020 season did not differ significantly (*p* = 0.246), while in the season 2020–2021, the difference becomes statistically significant (*p* < 0.05), with a hazard ratio of 5.14 (95% CI: 3.200 to 8.254).

## 4. Discussion

The main aim of this study was to determine whether professional soccer players who contracted SARS-CoV-2 had an increased risk of muscular injuries. The findings of this study reported a three to five times increased risk of muscle injury after SARS-CoV-2 infection.

Over the years, UEFA has conducted numerous studies on the match/minute ratio played by the elite athletes of the teams participating in its international tournaments to define a “normal” value for the incidence of injuries in football [22,23,24]. In particular, Hägglund et al.’s 11-year retrospective study [23] observed the incidence of injuries in more than 1000 h of play and found an incidence of 2 injuries per year per individual footballer or an average of 50 injuries if we take into consideration the team group (list of 25 athletes). If we consider only muscular injuries, the incidence drops to 15 injuries out of a shortlist of 25 footballers, with an average per athlete of 0.6 injuries [22]. These data are essential for commenting on the results of our study. Our results showed that in both leagues, players who did not contract COVID-19 had injury rates lower than the average of 0.6 reported by Bengtsson et al. [22] in both seasons. Instead, regarding players infected with SARS-CoV-2, they showed in the pre-COVID season an injury rate in line with the data of Bengtsson et al. [22], while in the 2020/2021 season, after the infection, it increased significantly in both leagues (1.44 for Serie A and 1.23 for LaLiga). The results relating to the change between seasons within individual groups showed significant differences in both groups; however, it should be specified that with such large samples, it is highly probable that even minimal variations between seasons are significant (especially in the C− group which is substantially larger than the C+ group), while if we look at the 95% confidence intervals we see an higher increase in number of injuries in the 2020/2021 season among football players who have had COVID-19. 

The results of minutes and matches played showed that in Serie A the players in the C+ group, during the 2019/2020 season, played more games and in total more minutes than the players in group C−; in the 2020/2021 season, the players in group C+ (which contracted COVID-19 during this season), they played fewer games and in total fewer minutes than in the previous season, on the contrary the players in group C− obtained an increase in total games and minutes played. This could be caused both by the period of absence linked to the infection itself and by the greater number of muscle injuries that players in group C+ have suffered, forcing them to have a longer period of absence from sporting activity. The same, albeit to a lesser extent, is observed in the Spanish league showing a substantial overlap of minutes played and games played in the 19/20 season between the two groups; however, in the following season, there was a reduction in minutes and games played in group C+ and an increase in games played and a slight reduction in minutes played in group C−. Our results indicate that SARS-CoV-2 infection could be a risk factor for muscular injury, in line with the recent study by Wezenbeek et al. [15]. Multiple linear regression analysis showed that in the 2019–2020 season, the risk of injury was significantly influenced by age, according to a previous study by Green et al. [25]. However, in the 2020–2021 season, SARS-CoV-2 infection was the variable that highly influenced the risk of muscle injury. Therefore, belonging to the C+ group in the 2020–2021 season completely distorts the correlation between the various variables and the muscle injury rates, becoming the main factor capable of influencing the latter. In other words, while older players with a high number of games and playing time were at greater risk of incurring muscle injuries in the 2019–2020 season, having contracted COVID-19 was the primary and most important risk factor in the following season. Similarly, in LaLiga, age was the main risk factor for muscle injury in the 2019–2020 season, while in the 2020–2021 season, the only variable that significantly influenced muscle injuries was the SARS-CoV-2 infection. The survival analysis showed that during the 2019–2020 season, the two groups had a similar risk of muscular injury. In the 2020/2021 season, the hazard risk ratio was 3.7 to 5.1 times greater among those athletes infected by SARS-CoV-2, in line with the results of Wezenbeek et al. [15], which reported a five-time higher hazard rate to develop a muscle strain after SARS-CoV-2 infection. One of the reasons that could explain the age effect only in the Italian league and not in the Spanish may depend on the fact that age is a variable not always correlated to injury. In fact, a recent review [26] reported that age influences athletes’ risk of calf muscle injuries, while another review [27] showed that age is not linked to a higher risk of quadriceps muscle injury. Nevertheless, the source of our data is not specific to the type of injury and therefore it was not possible to carry out a sub-analysis. Future studies will have to consider this element to draw definitive conclusions regarding the role of age on the risk of injuries.

As discussed by Wezenbeek et al. [15], there are two possible explanations for the correlation between SARS-CoV-2 infection and an increased risk of muscular injury. The first hypothesis is that strict quarantine rules implemented during the 2020/2021 season led to prolonged periods of abstention from training and sports participation, resulting in muscular detraining [28,29] and subsequent loss of muscle strength [30]. Muscle strength has been shown to be a protective factor against muscular injury [31], and the loss of muscle strength is more significant in highly trained athletes with greater initial muscle mass [32]. The second explanation is related to the direct biological effects of the virus. SARS-CoV-2 infection causes hyperinflammation, an increase in inflammatory markers, an increase in the neutrophil/lymphocyte ratio, and possible depletion of circulating T cells [33]. Furthermore, the virus may cause direct damage to muscle tissue by targeting the ACE 2 receptor [17], which is widely present in muscle tissue [34]. In addition, the virus can cause disturbances in blood flow and oxygen transport, leading to reduced muscle oxygenation during exercise. Lower muscle oxygen saturation [35] and VO_2_ max [36] are possible risk factors for muscle injury.

### 4.1. Clinical Implications

These results emphasize the importance of carefully considering the infection in the decision-making process for determining athletes’ readiness to return to sport (RTS). In fact, as already described by Elliot et al. [37] in addition to the known complications, it is necessary to consider that COVID-19 can be responsible for musculoskeletal complications. Therefore, our opinion is that the decision regarding the RTS should not only take into account the remission of COVID-19 related symptoms, rest and the cessation of post-COVID-19 drug therapy but should include specific assessment and training programs, generally used after a musculoskeletal injury [38] and therefore consider COVID-19 not only a risk factor but a real injury. 

### 4.2. Study Limitations

One possible limitation of this study is that the muscle injury events were collected from a single database, which may have resulted in some injuries being overlooked, omitted, or interpreted differently, leading to over- or underestimation. Another limitation of this study is that it was not possible to control for some factors that may have influenced the increased risk of injury, such as previous muscle injuries in unexamined seasons, severity of COVID-19-related symptoms, total period of quarantine and injured body part. Nevertheless, the study’s strength lies in its design, with a largely homogeneous population group of male professional soccer players followed prospectively with a standardized methodology.

## 5. Conclusions

This retrospective cohort study, conducted on a large sample of professional male football players from two main European leagues, has revealed a significant association between SARS-CoV-2 infection and increased risk of muscular injury. The results of this study show that the risk of muscular injury is 3.7 to 5.1 times higher in football players who have been infected with SARS-CoV-2. Overall, our study highlights the need for further investigation into the effects of SARS-CoV-2 on athletes and the development of tailored rehabilitation protocols to ensure a safe return to play.

## Figures and Tables

**Table 1:** Data collected and relative definitions used in the study.

Data	Description
Birth date	
Age at the end of the 2020–2021 season	
Team	
Muscle injuries season 2019–2020	Total number of muscular injury sustained by a player that resulted from a in-season football match or football training
Muscle injuries season 2020–2021	
Matches played in the 2019–2020	Total number of matches played during the season
Matches played in the 2020–2021	
Minutes played season 2019–2020	Total number of minutes played during the season
Minutes played season 2020–2021	
Date of SARS-CoV-2 infection	Date of in-season SARS-CoV-2 infection
Date of muscle injuries season 2019–2020	Date of each muscle injury occurred during in-season period
Date of muscle injuries season 2020–2021	
Season 2019–2020 start and end date	First and last match date of the season
Season 2020–2021 start and end date	

**Table 2:** Multiple linear regression analysis of Serie A football players. The factors described, relating to the 2019–2020 and 2020/2021 seasons, represent the dependent variables capable of influencing or not the number of muscle injuries within the same season.

Independent Variables	Coefficient	Std. Error	t	*p*	R^2^	Analysis of Variance	
						F-Ratio	*p*
2019/2020 season					0.041	7.5649	<0.05
(Constant)	−0.123						
Age	0.023	0.006	3.900	<0.05			
Minutes played	−0.000	0.000	−3.679	<0.05			
Matches played	0.025	0.007	3.613	<0.05			
Group (C+/C−)	−0.108	0.065	−1.658	0.098			
2020/2021 season					0.185	34.242	<0.05
(Constant)	1.770						
Age	0.020	0.007	2.814	0.005			
Minutes played	−0.000	0.000	−1.269	0.205			
Matches played	0.006	0.007	0.910	0.363			
Group (C+/C−)	−0.889	0.078	−11.371	<0.05			

**Table 3:** Multiple linear regression analysis of LaLiga football players. The factors described, relating to the 2019–2020 and 2020/2021 season, represent the dependent variables capable of influencing or not the number of muscle injuries within the same season.

Independent Variables	Coefficient	Std. Error	t	*p*	R^2^	Analysis of Variance	
						F-Ratio	*p*
2019/2020 season					0.007	1.113	0.349
(Constant)	0.110						
Age	0.0129	0.006411	2.022	0.044			
Minutes played	−0.000	0.00006924	−0.426	0.670			
Matches played	0.002	0.006129	0.337	0.736			
Group (C+/C−)	−0.042	0.06376	−0.663	0.508			
2020/2021 season					0.217	42.258	<0.05
(Constant)	2.141						
Age	0.000	0.007	0.087	0.931			
Minutes played	0.000	0.000	0.697	0.486			
Matches played	−0.004	0.006	−0.749	0.454			
Group (C+/C−)	−0.902	0.069	−12.916	<0.05			

## Data Availability

Data are available on reasonable request to the corresponding author.
